# Clustering of behavioural risk factors for health in UK adults in 2016: a cross-sectional survey

**DOI:** 10.1093/pubmed/fdy144

**Published:** 2018-09-06

**Authors:** Jack Birch, Robert Petty, Lucie Hooper, Linda Bauld, Gillian Rosenberg, Jyotsna Vohra

**Affiliations:** 1 Cancer Policy Research Centre (CPRC), Cancer Research UK, Angel Building, 407 St. John Street, London, UK; 2 Cancer Research UK, Angel Building, 407 St. John Street, London, UK; 3 University of Stirling, Stirling, UK

**Keywords:** alcohol consumption, obesity, physical activity

## Abstract

**Background:**

Foods high in fat, sugar and salt (HFSS) are known to contribute to overweight and obesity. In addition to overweight and obesity, smoking, alcohol consumption and physical inactivity are known risk factors for non-communicable diseases, including several cancers and cardiovascular disease.

**Methods:**

Secondary analysis of UK-representative cross-sectional survey data of 3293 adults aged 18+. Regression analyses were undertaken to understand the relationship between consumption of HFSS food and soft drinks, alcohol and tobacco and socio-demographics. Clustering analysis identified groupings of health risk factors.

**Results:**

Males, those aged 18–24 and those from the more deprived groups consumed ready meals and fast food most frequently. Most of the sample (77.3%) engaged in at least one health risk behaviour. Six clusters were identified in the clustering analysis. Older (65+) female respondents were more likely to be inactive. Smokers exhibiting additional risk behaviours were more likely to be of working age from more deprived groups, and men over 65 were more likely to consume harmful levels of alcohol with additional risk factors.

**Conclusion:**

Policies and services in the UK tend to focus on changing behaviour to address individual risk factors. This study shows that policies and interventions need to address multiple risk factors.

## Background

Health risk factors such as overweight/obesity, poor nutrition, smoking, alcohol consumption and low levels of physical activity each make a significant contribution to a number of non-communicable diseases (NCDs) including some types of cancer,^1–7^ diabetes^4,7,8^ and cardiovascular disease.^7–9^

Since the 1930s, policies have been developed regulate the availability of tobacco and alcohol products in the UK.^10^ Progress continues to be made to reduce the number of individuals who engage in tobacco or harmful alcohol use in the UK, such as through age and marketing restrictions, to minimize advertising exposure to young people.^10–12^ Smoking prevalence has fallen following policy interventions, such tobacco taxes, mass media campaigns and the introduction of smoke-free workplaces.^13^ England has seen general declines in alcohol consumption among young people.^14^ The introduction of minimum unit pricing in Scotland may encourage further declines in alcohol consumption, particularly among heavy drinkers.^15,16^

Fewer policies have been introduced to address rising levels of obesity in the UK, aside from the recently announced Soft Drink Industry Levy.^17^ This could be due to obesity being a complex health issue with no single contributing factor.^18^ McKinsey^19^ outlined some potential interventions. These measures included restrictions on the numbers of fast food outlets; food and drink reformulation; and restrictions on junk food advertising.^19^ One of the known major contributing factors to weight gain is consumption of foods high in fat, sugar and salt (HFSS), such as fast food,^20^ ready meals, soft drinks and confectionary.^21^ If implemented, these policies need to consider their contribution to health inequalities, as individuals living in areas of greater deprivation are more likely to have a higher BMI.^22^

Evidence has shown that populations who engage in multiple risk factors tend to have significantly worse health outcomes than those engaging in one health risk behaviour.^23^ Identifying groups of individuals whose health is affected by multiple risk factors provides insight to where policies need to be targeted to reduce inequalities in health.^24,25^

Clustering analysis identifies how different behaviours occur alongside each other and where in a particular population they may occur. Two recent systematic reviews have considered the clustering of multiple health risk factors. Noble *et al.*,^26^ took a global perspective, finding that more than half of studies found alcohol and smoking clustered together whilst a health found smoking, nutrition, alcohol and physical inactivity all clustered together. Meanwhile, Meader *et al.*,^27^ focused on literature from the UK. Meader *et al.*, found that alcohol and smoking consistently grouped in the study samples, and a strong association was also found between socioeconomic status and health risk behaviours. The principle limitation of the review was that the studies included were not representative of the UK population.

Studies which have considered consumption of HFSS food and drink as a behavioural risk factor have not previously considered this within a UK-wide population.^28–31^ This study aims to provide new data on this issue by describing the frequency of fast food and takeaways, ready meals, confectionary and soft drink consumption between population groups. Additionally, it examines the clustering of health risk behaviours: smoking, alcohol consumption, physical inactivity and overweight/obesity in adults in the UK to provide information that could inform more refined targeting of health policies and interventions.

## Methods

This study was a secondary analysis of data collected in February 2016 as previously described in Hooper *et al.*^32^ Data were collected from an online cross-sectional survey of 3490 adults aged 18 and over recruited by market research company, YouGov. A total of 3293 (94%) complete responses to the survey were received.

### Demographics

Demographic information for the respondents were held by YouGov and included: gender, age and region of residence (England, Scotland, Wales and Northern Ireland). Four groups from the National Readership Survey system for socio-economic status (SES) classification were used by YouGov. They were: AB (higher and intermediate managerial, administrative, professional occupations), C1 (supervisory, clerical and junior managerial, administrative and professional), C2 (skilled manual worker) and DE (semi-skilled and unskilled manual occupations, unemployed and lowest grade occupations).

Body mass index (BMI) was calculated from respondent self-reported weight and height (kg/m^2^). The following categories were used: underweight (<18.5 kg/m^2^), normal weight (18.5–24.9 kg/m^2^), overweight (25–29.9 kg/m^2^) and obese (>30 kg/m^2^).^33^ Underweight and normal were coded as not having weight as a health risk factor, while the overweight and obese categories were coded as having the risk factor.

### Health behaviours

Questions were asked about four additional health behaviours: diet, smoking, alcohol and physical activity.

#### Diet

Consumption of food high in fat was estimated by asking about the consumption frequency of fast food and ready meals. Questions asked were: ‘How often do you have food….—From takeaway places like McDonalds, Burger King, Pizza Hut, KFC or local takeaway food places?’ and ‘How often do you have food….—At home such as ready meals, burgers, pizza, or chips?’. Participants could answer: two to three times a day; once a day, 5–6 times a week; 2–4 times a week; once a week; 1–3 times a month; < once or a month; and never. Responses were coded as either ‘at least once a week’ or ‘less than once a week’ for each category.

The consumption of sugar-sweetened beverages and food that are high in sugar was estimated by asking: ‘How often do you …—Drink soft drinks such as cola, cordials, sports drinks or energy drinks (do not include sugar free drinks)?’ and ‘How often do you …—Eat confectionery (such as sweets and chocolates), cakes, muffins, sweet pies, pastries or biscuits?’. Participants could answer: >6 times a day; 4–5 times a day; 2–3 times a day; once a day; 5–6 times a week; 2–4 times a week; once a week; 1–3 times a month; < once a month; and never. Responses were coded as ‘once a day or more’ and ‘less than once a day’.

#### Smoking

Smoking status was defined from: ‘I have never smoked’; ‘I used to smoke but haven given up now’; ‘I smoke but I don’t smoke every day’ and ‘I smoke every day’. The first two responses were coded as not having smoking as a risk factor; those who gave the latter two responses were coded as having smoking as a risk factor, in line with published studies.^34,35^

#### Alcohol consumption

Weekly alcohol consumption was estimated by asking ‘how often do you have a drink containing alcohol?’ and ‘how many units of alcohol do you drink on a typical day when you are drinking?’. The respondents were then classified as either ‘low-risk drinkers’ (consume 14 or less units of alcohol per week) or ‘increased-risk drinkers’ (more than 14 units per week), with those in the latter category coded as having alcohol consumption as a risk factor. These categories were based on current UK Chief Medical Officers’ recommendations on low-risk drinking.^36^

#### Physical activity

Physical activity level questions were taken from the short-form International Physical Activity Questionnaire (IPAQ).^37^ Respondents were asked three questions—for how many hours and minutes did they partake in: vigorous activity, moderate activity and walking. Respondents were classed as ‘Inactive’, ‘Minimally active’ or ‘Highly active’. For analysis, the latter two categories were merged, and those who were ‘Inactive’ were coded as having physical inactivity as a risk factor.

All risk factors were coded as 0 for no risk and 1 for having that risk factor.

### Analysis

IBM SPSS Version 24 was used to analyse the data. Age, gender, socioeconomic status and region were weighted to ensure the results were representative of the population. The weighting was provided by YouGov and counteracted the under-representation of those aged 18–34, residents from England and those from a DE socioeconomic group (as shown in Supplementary Table SI). Unless specified, weighted results are presented.

Cross-tabulations were undertaken to produce descriptive statistics between the diet, alcohol and smoking risk factor variables and the demographic variables. Binary logistic regression—chi-square analysis—was performed to explore relationships between the risk factor variables.

Clustering analysis was performed on unweighted data to identify behaviour patterns. Health risk factors included in the clustering analysis were: smoking, alcohol consumption, physical inactivity and overweight/obesity (BMI). BMI was included as a proxy for diet as there is little indication in the literature around what level of consumption of ready meals, takeaways, soft drinks or confectionary constitutes a risk to health.

As the sample size was more than 1000 participants, the Two-Step Cluster method was chosen.^38^ Automated cluster selection was used to determine the number of clusters formed. An average silhouette coefficient was produced to determine how well each case within a cluster matched to each other and how separate each cluster was from the other clusters.^38^ Additional regression analyses were conducted to test for associations between clusters and demographic variables.

## Results

Data for gender, age, nation of residence and socioeconomic status were available for all 3293 respondents. A total of 259 respondents (7.8%) of the unweighted sample (290 (8.8%) of the weighted sample) did not provide height and weight details, so their BMI could not be calculated. These cases were subsequently excluded from the analysis. See Supplementary Table SI for further details.

Males were significantly more likely to consume ready meals and fast food at least once a week, consume soft drinks at least once a day, and consume more than 14 units of alcohol per week. Females were significantly more likely to be physically inactive (shown in Tables 1 and 2).

**Table 1:** Multiple regression of convenience food and sugar-sweetened soft drinks

	Ready meals			Fast food and takeaways			Confectionary			Soft drinks		
	At least once a week (%)	OR	CI	At least once a week (%)	OR	CI	At least once a day (%)	OR	CI	At least once a day (%)	OR	CI
Total	**49.9**			**16.0**			**17.4**			**40.4**		
Gender												
Male (*n* = 1604)	55.6	–	–	19.3	–	–	42.0	–	–	20.3	–	–
Female (*n* = 1690)	**44.4**	0.603**	**0.519–0.700**	**12.9**	0.602**	**0.489–0.741**	39.0	0.936	0.807–1.086	**14.7**	0.644**	**0.526–0.788**
Age												
18–24 (*n* = 283)	61.1	–	–	27.6	–	–	49.1	–	–	23.0	–	–
25–34 (*n* = 631)	54.5	0.780	0.573–1.062	26.3	1.052	0.743–1.491	45.2	0.835	0.631–1.152	23.3	1.103	0.755–1.612
35–44 (*n* = 571)	56.6	0.802	0.584–1.101	21.2	0.728	0.504–1.053	43.5	0.795	0.584–1.083	22.4	0.979	0.662–1.448
45–54 (*n* = 562)	54.4	0.736	0.533–1.014	**14.9**	0.460**	**0.310–0.683**	**38.1**	0.653*	**0.476–0.895**	20.5	0.795	0.530–1.190
55–64 (*n* = 510)	**45.5**	0.480**	**0.347–0.664**	**9.4**	0.269**	**0.174–0.415**	**32.9**	0.521**	**0.377–0.720**	**10.4**	0.368**	**0.235–0.578**
65+ (*n* = 736)	**35.7**	0.326**	**0.239–0.444**	**4.2**	0.098*	**0.059–0.162**	**37.6**	0.635*	**0.470–0.858**	**8.7**	0.309**	**0.201–0.476**
Social grade												
AB (*n* = 725)	47.2	–	–	13.1	–	–	41.6	–	–	14.9	–	–
C1 (*n* = 988)	49.4	0.972	0.789–1.198	18.1	1.175	0.872–1.583	43.0	1.009	0.820–1.241	16.3	0.892	0.664–1.197
C2 (*n* = 494)	**54.3**	1.359*	**1.063–1.738**	**19.0**	1.681*	**1.195–2.364**	41.5	1.012	0.793–1.292	19.0	1.266	0.906–1.769
DE (*n* = 1087)	50.1	1.198	0.975–1.471	14.6	1.237	0.913–1.677	36.8	0.869	0.708–1.067	**19.3**	1.370*	**1.034–1.816**
BMI												
Underweight (*n* = 85)	49.4	–	–	16.5	–	–	35.3	–	–	23.5	–	–
Normal weight (*n* = 1327)	49.3	0.953	0.606–1.498	14.6	0.858	0.462–1.594	42.7	1.418	0.891–2.254	**12.8**	0.444*	**0.258–0.763**
Overweight (*n* = 944)	46.9	0.910	0.574–1.442	14.8	1.096	0.583–2.060	39.5	1.321	0.823–2.118	15.4	0.612	0.353–1.063
Obese (648)	57.3	1.416	0.886–2.264	20.7	1.719	0.909–3.250	38.3	1.278	0.790–2.069	25.3	1.154	0.664–2.005
Nation												
England (*n* = 2762)	49.1	–	–	15.5	–	–	39.3	–	–	16.4	–	–
Wales (*n* = 158)	55.1	1.098	0.773–1.558	18.4	1.045	0.663–1.645	43.0	1.050	0.744–1.480	24.7	1.391	0.912–2.122
Scotland (*n* = 280)	52.1	1.025	0.787–1.336	16.1	0.910	0.632–1.309	**48.2**	1.438*	**1.110–1.863**	21.1	1.278	0.919–1.777
Northern Ireland (*n* = 92)	56.5	1.261	0.805–1.975	**26.1**	1.796*	**1.066–3.025**	46.7	1.311	0.848–2.025	23.9	1.617	0.957–2.733

SES: AB is managerial/professional roles, C1 supervisory and clerical roles, C2 skilled manual workers, DE semi-skilled, unskilled and unemployed.

*P < 0.05, **P < 0.005.

**Table 2:** Multiple regression of tobacco, alcohol and physical inactivity risk factors

	Tobacco use			Alcohol consumption			Physically inactive		
	Current smoker (%)	OR	CI	Consume >14 units per week (%)	OR	CI	Not minimally active (%)	OR	CI
Total	**14.6**			**15.3**			**33.9**		
Gender									
Male (*n* = 1604)	15.8	–	–	23.1	–	–	29.0	–	–
Female (*n* = 1690)	13.6	0.826	0.673–1.013	**7.9**	0.292**	**0.233–0.365**	**38.6**	1.584**	**1.348–1.852**
Age									
18–24 (*n* = 283)	16.3	–	–	9.9	–	–	20.5	–	–
25–34 (*n* = 631)	14.6	0.932	0.622–1.397	11.0	0.993	0.614–1.607	**30.4**	1.641*	**1.123–2.398**
35–44 (*n* = 571)	17.5	1.124	0.747–1.692	13.5	1.314	0.813–2.125	**33.8**	1.851*	**1.261–2.718**
45–54 (*n* = 562)	15.8	0.936	0.632–1.465	**18.3**	1.976*	**1.229–3.178**	**37.5**	2.104**	**1.430–3.095**
55–64 (*n* = 510)	17.6	1.045	0.686–1.592	**19.7**	2.137*	**1.326–3.443**	**31.8**	1.634*	**1.103–2.422**
65+ (*n* = 736)	**8.8**	0.508*	**0.330–0.782**	**17.0**	1.757*	**1.104–2.795**	**40.9**	2.529**	**1.746–3.663**
Social grade									
AB (*n* = 725)	10.2	–	–	17.7	–	–	32.0	–	–
C1 (*n* = 988)	12.2	1.195	0.869–1.645	17.4	1.084	0.824–1.425	29.9	0.915	0.728–1.151
C2 (*n* = 494)	**17.0**	1.757*	**1.238–2.492**	15.6	0.886	0.638–1.232	34.8	1.113	0.855–1.449
DE (*n* = 1087)	**18.6**	2.046**	**1.517–2.758**	**11.6**	0.675*	**0.508–0.897**	38.5	1.182	0.949–1.472
BMI									
Underweight (*n* = 85)	9.9	–	–	10.6	–	–	37.6	–	–
Normal weight (*n* = 1327)	16.9	1.810	0.869–3.770	14.2	1.230	0.590–2.566	**25.5**	0.562*	**0.351–0.897**
Overweight (*n* = 944)	12.4	1.284	0.607–2.713	19.1	1.383	0.659–2.903	28.8	0.655	0.407–1.056
Obese (648)	16.0	1.623	0.765–3.447	15.0	1.168	0.549–2.487	**54.3**	1.859*	**1.149–3.007**
Nation									
England (*n* = 2762)	14.4	–	–	15.6	–	–	33.5	–	–
Wales (*n* = 158)	15.2	1.069	0.673–1.699	12.7	0.925	0.558–1.533	40.5	1.358	0.943–1.954
Scotland (*n* = 280)	14.3	0.908	0.628–1.312	13.9	0.882	0.608–1.280	31.5	0.854	0.638–1.143
Northern Ireland (*n* = 92)	20.7	1.465	0.856–2.508	12.0	0.693	0.352–1.366	42.4	1.559	0.987–2.462

SES: AB is managerial/professional roles, C1 supervisory and clerical roles, C2 skilled manual workers, DE semi-skilled, unskilled and unemployed.

*P < 0.05, **P < 0.005.

Younger individuals aged 18–24 were more likely to consume: ready meals at least once a week compared to respondents aged 55–65+; fast food at least once a week and confectionary at least once a day compared to those aged 45–65+; soft drinks at least once a day compared to those aged 55–65+ and be more likely to be a current smoker than adults aged 65+. Individuals aged 18–24 were less likely to consume more than 14 units of alcohol in a week than those aged 45–65+; and were less likely to be physically inactive compared to all other age groups (Tables 1 and 2).

Differences also exist between socioeconomic groupings in relation to consumption behaviours. Individuals in the managerial (AB) category were less likely to consume ready meals at least once a week compared to skilled manual workers (C2); consume fast food and takeaways at least once a week than skilled manual workers (C2); consume soft drinks at least once a day relative to unskilled/unemployed (DE) individuals; and were more likely to smoke than skilled manual workers (C2) or unskilled/unemployed (DE) individuals. Meanwhile, individuals in the managerial (AB) group were more likely to consume more than 14 units of alcohol per week compared to unskilled/unemployed (DE) individuals.

Respondents from England were less likely to consume fast food and takeaways at least once a week than those from Northern Ireland, and less likely to consume confectionary at least once a day than those from Scotland.

### Risk factors

**Fig. 1:**
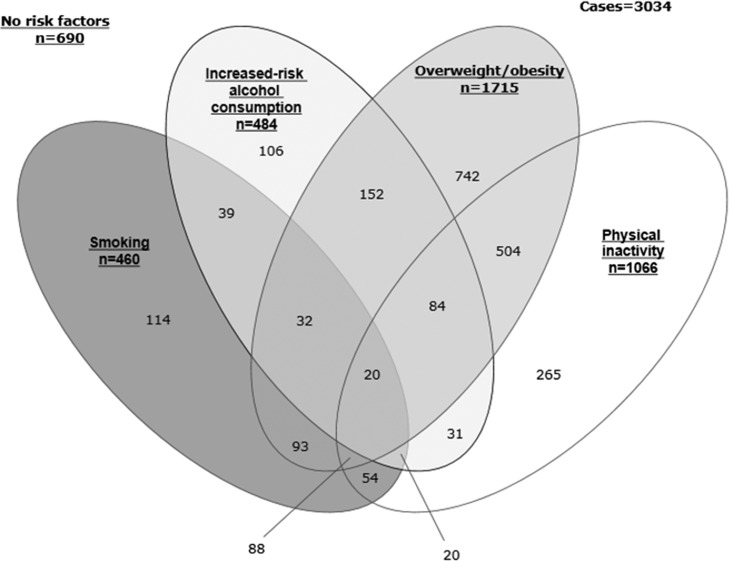
Distribution of risk factors.

Unweighted data were used to consider the distribution of risk factors. Of the 3034 eligible participants: 690 (22.7%) presented none of the risk factor behaviours; 1227 (40.4%) presented a single risk factor; 873 (28.8%) presented two; 224 (7.4%) presented three; and 20 (0.7%) presented all four risk factor behaviours (Fig. 1).

### Clustering of risk factors

A total of 3034 cases were included in the cluster analysis; 259 cases were excluded prior to the cluster analysis as BMI could not be calculated due to height and weight data not being provided. This analysis produced six clusters. The average silhouette measure of cohesion and separation was 0.8, demonstrating the quality of the clusters is good. The ratio of the largest cluster (cluster 2) to the smallest cluster (cluster 6) was 2.80:1. Descriptions of the clusters are outlined below:

#### Cluster 1: No risk factors

A total of 690 individuals (22.7%) presenting no risk factors made up this cluster. In total, 32.7% were males. Of the different age categories: 13.9% were 18–24, 27.2% were 25–34, 15.8% were 35–44, 11.7% were 45–54, 12.4% were 55–64 and 19.0% were 65+. This cluster was used as the reference category in the regression analysis.

#### Cluster 2: Overweight/obese, otherwise low risk

This cluster included 742 (24.5%) individuals, all exclusively only having overweight/obese as a risk-factor. Those in this cluster are: more likely to be male and be aged 65+ than 18–24 or 25–34.

#### Cluster 3: Inactive and overweight/obese

All individuals (504, 16.6%) only had physical inactivity and overweight/obese risk factors present. Both age and socioeconomic differences are present in this cluster. Individuals are more likely to be aged 65+ than 18–24, 25–34 or 35–44, and are more likely to belong to the C2 socioeconomic category than DE.

#### Cluster 4: Multiple risk factors (increased-risk alcohol)

In this cluster of 484 (16.0%) cases, all cases were in the increased-risk category for alcohol, 59.5% were overweight/obese, 32% were physically inactive and 22.9% smoked. Individuals in this cluster are more likely to be male and aged 65+ rather than 18–24 or 25–34.

#### Cluster 5: Multiple risk factors (smoking)

Overall, 349 (11.5%) individuals made up this cluster. All the individuals smoked, 51.9% were overweight or obese and 40.7% were physically inactive. None of the individuals in this cluster had alcohol consumption as a risk factor. Individuals in this cluster were more likely to be in the 35–44, 45–54 or 55–64 age categories than in the 65+ category. Socioeconomic differences were also present; individuals are more likely to belong to the DE category than to either the AB or C1 categories.

#### Cluster 6: inactive

This cluster contained 265 (8.7%) cases, all of which only had physical inactivity as a risk factor. Individuals are more likely to be female, and be aged 65+ rather than 18–24, 25–34 and 55–64 (shown in full in Table 3).

**Table 3:** Multiple regression of the cluster variable

	Healthy, low risk (reference)		Overweight/obese, otherwise low risk		Inactive and overweight/obese		Multiple risk factors (Increased-risk alcohol)		Smoking		Physically inactive	
	Cluster 1		Cluster 2		Cluster 3		Cluster 4		Cluster 5		Cluster 6	
	OR	CI	OR	CI	OR	CI	OR	CI	OR	CI	OR	CI
Gender												
Male (*n* = 1604)			1.487**	**1.205–1.834**	0.943	0.740–1.203	3.830**	**2.968–4.942**	1.287	0.992–1.669	0.643*	**0.481–0.860**
Female (*n* = 1690)			–	–	–	–	–	–	–	–	–	–
Age												
18–24 (*n* = 283)			0.293**	**0.189–0.455**	0.118**	**0.062–0.225**	0.284**	**0.172–0.467**	0.921	0.557–1.522	0.576*	**0.344–0.966**
25–34 (*n* = 631)			0.477**	**0.345–0.660**	0.328**	**0.227–0.475**	0.352**	**0.240–0.515**	0.927	0.604–1.424	0.641*	**0.423–0.972**
35–44 (*n* = 571)			0.913	0.651–1.279	0.554*	**0.376–0.819**	0.692	0.469–1.022	1.572*	**1.009–2.450**	1.231	0.807–1.876
45–54 (*n* = 562)			1.343	0.947–1.905	1.057	0.724–1.542	1.332	0.904–1.964	1.779*	**1.122–2.822**	1.012	0.632–1.621
55–64 (*n* = 510)			1.117	0.786–1.588	0.887	0.606–1.298	1.246	0.849–1.830	1.876*	**1.197–2.941**	0.411*	**0.230–0.734**
65+ (*n* = 736)			–	–	–	–	–	–	–	–	–	–
Social grade												
AB (*n* = 725)			0.958	0.717–1.279	1.043	0.755–1.442	1.315	0.944–1.833	0.465**	**0.319–0.678**	0.798	0.539–1.183
C1 (*n* = 988)			1.004	0.768–1.312	0.829	0.604–1.137	1.410	1.032–1.927	0.528**	**0.379–0.736**	0.831	0.583–1.186
C2 (*n* = 494)			1.339	0.983–1.881	1.479*	**1.017–2.150**	1.620	1.094–2.398	1.043	0.705–1.543	1.435	0.936–2.200
DE (*n* = 1087)			–	–	–	–	–	–	–	–	–	–
Nation												
England (*n* = 2762)			0.833	0.422–1.644	0.555	0.273–1.129	1.038	0.465–2.320	0.491	0.241–0.998	0.673	0.297–1.526
Wales (*n* = 158)			1.117	0.485–2.569	1.193	0.502–2.838	1.267	0.475–3.382	0.639	0.257–1.577	0.749	0.267–2.103
Scotland (*n* = 280)			1.085	0.511–2.301	0.569	0.252–1.286	0.982	0.401–2.403	0.487	0.213–1.111	0.533	0.205–1.386
Northern Ireland (*n* = 92)			–	–	–	–	–	–	–	–	–	–

SES: AB is managerial/professional roles, C1 supervisory and clerical roles, C2 skilled manual workers, DE semi-skilled, unskilled and unemployed.

* *P* < 0.05, **P < 0.005

## Discussion

This study used a UK-wide population representative sample to measure ready meal, fast food and takeaway, confectionary and soft drink consumption and to assess the clustering of preventable risk factors for NCDs. Males reported more frequent consumption of ready meals, fast food and takeaways and soft drinks than females. This is consistent with previous research indicating females are more likely to avoid energy dense foods.^39^ Despite this, males were less likely to be physically inactive, consistent with global trends.^40^

Socioeconomic differences existed across consumption behaviours as those from lower socioeconomic categories were more likely to consume convenience foods and be a current smoker than those from the highest socioeconomic group (AB). As there are a higher proportion of fast food outlets in areas of socioeconomic deprivation in the UK,^41^ this may provide an environment for those who are more deprived to consume more food that is HFSS than those who are less deprived. Previous data have shown that those who live closer to fast food outlets are known to consume more fast food and are likely to have a higher BMI.^20^

Clustering analysis was performed to identify groups of individuals within the population that engage in behaviours or have a BMI level which is overweight/obese that impacts on mortality and morbidity. We sought to identify populations who engage in multiple health risk behaviours and have overweight/obese BMI that could be at greatest need of targeted public health interventions. Six clusters were formed, with some similarity between groups, especially for clusters 4 and 5. Individuals in these groups exhibit multiple risk factors and represent the greatest potential for targeted health policies. Cluster 4 (Multiple risk factors (increased-risk alcohol), 16.0%, *n* = 484) contained respondents who were at increased-risk alcohol consumption as a risk factor, supporting data from the Office for National Statistics which suggests older individuals are more likely to drink more frequently.^42^ Respondents in cluster 5 (Multiple risk factors (smoking)) show similar characteristics to those in cluster 4, with the main behavioural differences being that all respondents in cluster 5 smoked, but none had increased-risk alcohol consumption as a risk factor. The differences between clusters 4 and 5 could be explained by demographic differences. Those in cluster 4 were more likely to be male and be of retirement age (65+ rather than 18–24 or 25–34) compared to individuals with no risk factors (cluster 1). Individuals in cluster 5 were more likely to be of working age (35–64 than 65+) and be classified in a lower socioeconomic group (DE versus AB or C1). This is consistent with previous research which has found that the most disadvantaged were more likely to smoke than the most advantaged.^43,44^

Cases from clusters 2–5 were more likely to be male, and generally had an age profile indicative of being at least in mid-life. Almost regardless of whichever risk factor an individual had and what that co-occurred with, older men appeared to be more likely to have all the health risk behaviours. This is contradictory to Noble *et al.* and Meader *et al.*, who found ambiguous associations between gender, age and behavioural clustering. One cause could be limited health knowledge, so novel strategies may be effective in engaging older males, increasing awareness and improving health behaviours.^45^ Similarly, other factors not included in this study such as partnership status^35,46,47^ may contribute to the association.

### What is already known about the topic

Recent data on consumption of sugar-sweetened soft drinks in the UK have been collected in the National Diet and Nutrition Survey.^48^ However, few data exist around the consumption of convenience foods such as ready meals and takeaways, and sweet confectionary items. Data on these can improve understanding of dietary factors that contribute to overweight and obesity.

Systematic reviews have been conducted into the clustering or co-occurrence of health risk behaviours. One took a worldwide perspective,^26^ whilst the other focused on literature solely from the UK.^27^ The latter review found that most studies considered clustering between two health risk factors; few considered three or more risk factors. Most of the current clustering literature considers clustering between only two risk factors.^27^ Some studies have considered the clustering of health risk behaviours amongst different adult populations within the UK,^35,49–51^ while only a single study used a UK-wide sample.^52^ However, whilst this previous study examined four risk factors, only two were general lifestyle risk factors—smoking and sugar consumption. The remaining two risk factors studied were specific to dental hygiene.

### What the study adds

This study is the first to assess UK-wide consumption of ready meals, fast food and takeaways, and sweet confectionary to better understand dietary factors that may contribute to overweight and obesity. Additionally, the study considers the clustering of multiple health risk behaviours as well as BMI across a UK-wide population representative sample. Previously using BMI as a risk factor in this context has only been performed in a non-UK sample.^53^

### Limitations of the study

The nature of secondary data analysis means available variables may limit the analyses that can be conducted, and comparisons made. For example, no data were collected about fruit and vegetable consumption. As much of the existing literature identifies consuming less than five portions as having a poor diet,^35,50,51^ it may limit the comparisons between this and other studies. The addition of long term outcome data would increase validity to the findings of this study by furthering understanding of how lifestyle choices affect health, but this was beyond the scope of this study.

Whilst the weighted data ensured that the results of the consumption variables analyses were population representative, unweighted data were used for the clustering analysis. Therefore, the results of the clustering analysis may not be truly representative of the UK population. A further limitation of the clustering analyses was the binary coding of variables, as this approach may have prevented certain groupings from emerging in the clustering analysis.

The risk behaviours and BMI data used in the analyses were self-reported by the participants. This could explain why no association was observed between diet and BMI, as respondents may have underestimated either their weight or diet leading to an insignificant association to be observed. Similarly, as the data were cross-sectional they may only reflect the moment in time they were collected.

### Implications

Those from more deprived groups are more likely to consume HFSS foods than those from less deprived groups, yet fast food outlet density is highest in deprived areas. Planning laws could be better applied to consider the make-up and health of the local population. Further, older people and those indicating multiple risk factor behaviours are at highest risk of diseases such as heart disease and some cancers. Policies and interventions may need to better target populations at highest risk; including consideration of how to address the clustering of multiple risk factors in particular populations.

This study found that certain groups in the population, especially older males, are at risk from several health risk factors such as smoking, excessive alcohol consumption, physical inactivity and overweight/obesity. Therefore, policies need to target this particular group to prevent the occurrence of many NCDs.

## Supplementary Material

fdy144_Demographics_table
