# Supplementary material for: Clustering of behavioural risk factors for health in UK adults in 2016: a cross-sectional survey
Source: J Public Health (Oxf). 2018 Sep 6;41(3):e226–36. doi: 10.1093/pubmed/fdy144 (PMC6785700; doi:10.1093/pubmed/fdy144)
Supplement: fdy144_Demographics_table [file fdy144_demographics_table.docx]

Supplementary table: Sample demographics

|  | Unweighted Sample (n=3293) | Weighted Sample (n=3293) |
| --- | --- | --- |
|  | n (%) | n (%) |
| Gender | | |
| Male | 1580 (48) | 1604 (48.7) |
| Female | 1713 (52) | 1689 (51.3) |
| Age | | |
| 18-24 | 194 (5.9) | 283 (8.6) |
| 25-34 | 494 (15.0) | 631 (19.2) |
| 35-44 | 639 (19.4) | 571 (17.3) |
| 45-54 | 627 (19.0) | 562 (17.1) |
| 55-64 | 596 (18.1) | 510 (15.5) |
| 65+ | 743 (22.6) | 737 (22.4) |
| Nation of Residence | | |
| England | 1773 (53.8) | 2762 (83.9) |
| Wales | 503 (15.3) | 158 (4.8) |
| Scotland | 513 (15.6) | 280 (8.5) |
| Northern Ireland | 504 (15.3) | 92 (2.8) |
| Socioeconomic status (SES) | | |
| AB | 913 (27.7) | 724 (22.0) |
| C1 | 1037 (31.5) | 988 (30.0) |
| C2 | 538 (16.3) | 494 (15.0) |
| DE | 805 (24.4) | 1087 (33.0) |
| Body mass index (BMI) | | |
| Underweight | 75 (2.3) | 85 (2.6) |
| Normal Weight | 1244 (37.8) | 1327 (40.3) |
| Overweight | 1015 (30.8) | 944 (28.7) |
| Obese | 700 (21.3) | 648 (19.7) |
| Not calculated | 259 (7.9) | 290 (8.8) |
